# MDM4 at the Crossroads: Beyond p53 and MDM2

**DOI:** 10.3390/cancers18071059

**Published:** 2026-03-25

**Authors:** Dipesh Thapa, Allison St. John, Alejandro Parrales, Atul Ranjan, Tomoo Iwakuma

**Affiliations:** 1 Section of Hematology, Oncology, and Bone Marrow Transplant, Department of Pediatrics, Children’s Mercy Research Institute, Kansas City, MO 64108, USA; dthapa@cmh.edu (D.T.); astjohn@cmh.edu (A.S.J.); aparralesbriones@cmh.edu (A.P.); aranjan@cmh.edu (A.R.); 2 Department of Cancer Biology, University of Kansas Medical Center, Kansas City, KS 66160, USA

**Keywords:** MDM4, MDM2, p53, tumorigenesis, independent, ferroptosis, replication fork progression

## Abstract

MDM4 (Murine Double Minute 4), also known as MDMX, is a crucial negative regulator of the tumor suppressor p53. MDM4 heterodimerizes with MDM2 to enhance MDM2-mediated ubiquitination and degradation of p53, thereby promoting tumorigenesis. Beyond its canonical role in inhibiting p53 activity, recent studies have revealed diverse p53-independent functions. MDM4 interacts with various proteins, including p73, E2F1, casein kinase 1α, PPARα, and TRIM21 to regulate cell cycle progression, β-catenin-mediated pre-leukemic progression, and ferroptosis independent of p53. In addition, MDM4 functions independently of both p53 and MDM2 by interacting with proteins, such as SMAD family members 3/4, retinoblastoma protein (pRB), p21, Nbs1 (also known as Nibrin), mTOR complex 1 (mTORC1), and the Polycomb Repressive Complexes (PRCs) complex, to control cell proliferation and survival, as well as protein degradation, double-strand break (DSB) repair, and replication fork progression. Intriguingly, multiple studies suggest that MDM4 exhibits oncogenic activity independent of p53; however, other reports highlight a potential tumor-suppressive role for MDM4 in the absence of p53. Thus, MDM4’s functions extend well beyond the canonical p53–MDM2 axis. A deeper understanding of MDM4 biology may facilitate the development of novel targeted therapies for various cancers.

## Simple Summary

MDM4 (also called MDMX) is a protein that helps control how cells grow and respond to stress. It is best known for reducing the activity of p53, a major “guardian” protein that protects cells from becoming cancerous. MDM4 often works with another protein, MDM2, to lower p53 levels. However, MDM4 also has several other important roles beyond p53, and in some cases, these are independent of MDM2 as well. It helps regulate how quickly cells divide, how they repair damaged DNA, how they respond to stress, and how safely they copy their DNA before dividing. Because of these many roles, MDM4 can sometimes promote cancer growth, yet in other situations—especially when p53 is missing—it can slow cancer development. Understanding these diverse functions makes MDM4-associated pathways and interacting proteins potential therapeutic targets for cancers with dysregulated MDM4 signaling.

## 1. Introduction

Mdm4 was originally identified as a protein that binds to bacterially synthesized human tumor suppressor p53 through plaque hybridization screening of *Escherichia coli* infected with a bacteriophage library containing cDNAs derived from whole mouse embryos [1,2]. The identified cDNA showed high homology to Mdm2 cDNA and protein and was therefore named MdmX, later referred to as Mdm4. Like Mdm2, Mdm4 binds to p53 and inhibits its transcriptional activity [1,2,3]. Genetic deletion of *Mdm4* in mice resulted in mid-gestation embryonic lethality due to proliferative arrest, which was fully rescued by concomitant *p53* deletion, functioning similarly to Mdm2 [3,4,5]. In lymphoma mouse models, in which tumors from *Mdm4^+/+^p53^KI/−^EµMyc* and *Mdm4^−/−^p53^KI/−^EµMyc* mice were transplanted, induction of p53 (p53ER^TAM^) with seven days of tamoxifen treatment significantly increased overall survival in mice bearing *Mdm4^−/−^p53^KI/−^EµMyc* lymphomas compared with those bearing *Mdm4^+/+^p53^KI/−^EµMyc* lymphomas [6]. These findings established MDM4 as a key negative regulator of p53.

Clinically, MDM4 overexpression has been observed in approximately 20% of all human cancers, mainly due to gene amplification, including acute myeloid leukemia (AML, 92%), myelodysplastic syndrome (52%), Ewing sarcoma (50%), synovial sarcoma (44%), and osteosarcoma (35%) [7,8,9]. MDM4 overexpression has been correlated with poor prognosis across multiple cancers, including soft-tissue sarcoma [10], chronic lymphocytic leukemia (CLL) [11], and AML [12]. These clinical observations strongly support the oncogenic functions of MDM4. 

**Figure 1:**
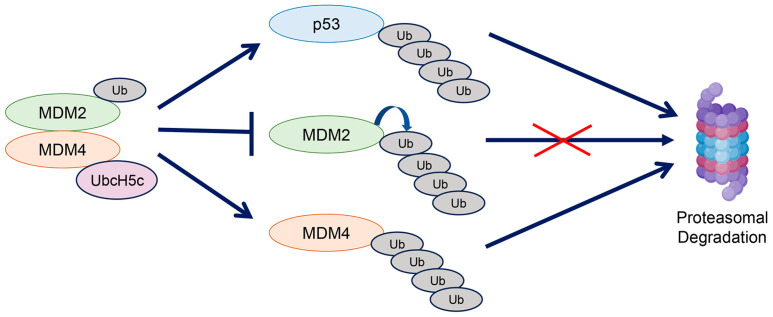
Canonical functions of MDM4. MDM4 binds to MDM2 and enhances its E3 ubiquitin ligase activity to promote p53 degradation, while simultaneously preventing MDM2 self-ubiquitination and subsequent degradation (denoted by the red cross). Conversely, MDM2 can ubiquitinate and degrade MDM4 in a context-dependent manner.

Mechanistically, MDM4 inhibits p53 activity, mainly by forming a heterodimer with MDM2 to enhance the MDM2-mediated ubiquitination of p53 [13,14,15]. The MDM2-MDM4 heterodimer also decreased MDM2 self-ubiquitination and stabilized MDM2, while increasing the ubiquitination and degradation of MDM4 in a context-dependent manner [13,14] (Figure 1). In addition to its effects on ubiquitination, MDM4 enhanced the MDM2-mediated p53 neddylation by forming a heterodimer, which potentially led to reduced p53 function [16,17]. Similarly to MDM2, MDM4 reduced p300/CBP-mediated p53 acetylation, thereby attenuating p53 activity. However, whether this function depends on MDM2 remains unclear [18,19].

The C-terminal RING domain of MDM4 mediates the formation of a stable heterodimer with the RING domain of MDM2 (Figure 1), resulting in more efficient p53 degradation than that achieved by MDM2 homodimers [13,20]. MDM4 cannot control the protein stability of p53 on its own in the absence of MDM2, due to the lack of E3 ubiquitin ligase activity in the MDM4 RING domain [21]. Instead, MDM4 recruits UbcH5c, an E2 conjugating enzyme, through the C-terminal domain to facilitate MDM2-dependent p53 ubiquitination [22]. The significance of the Mdm2-Mdm4 heterodimer was also demonstrated by in vivo studies using a *Mdm4^C462A/C462A^* mouse model, which disrupted Mdm2-Mdm4 binding. The *Mdm4^C462A/C462A^* mice exhibited embryonic lethality that was rescued by the p53 deletion, reminiscent of *Mdm4^−/−^* mice [23]. Similarly, deletion of the Mdm4 RING domain in mice caused p53-dependent embryonic lethality [24]. Thus, the ability of MDM4 to heterodimerize with MDM2 through the RING domain is crucial for suppressing the p53 activity. These two in vivo studies further reinforce the MDM2-dependent function of MDM4 in suppressing p53 activity. 

**Figure 2:**
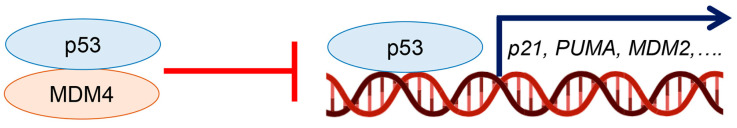
MDM2-independent inhibition of p53 activity by MDM4. MDM4 directly binds to p53 and inhibits its transcriptional activity. “….” denotes additional downstream target genes of p53.

Mdm4 was shown to bind p53 independent of Mdm2 using in vitro-translated Mdm4 and bacterially produced p53 proteins [2]. Crystal structure analysis of the N-terminal domain of MDM4 bound to a 15-residue N-terminal p53 peptide (amino acids 15–29) revealed that the MDM4–p53 complex closely resembled the MDM2–p53 complex. However, the hydrophobic cleft of MDM4 differed from that of MDM2, explaining the inefficiency of an MDM2 antagonist, Nutlin-3a, in disrupting the MDM4–p53 interaction [25]. Using conditional induction of p53 in *p53^LSL/−^* mouse embryonic fibroblasts (MEFs) with different *Mdm2* and *Mdm4* genotypes, Mdm4 was shown to functionally inhibit p53 activity in the absence of Mdm2 [26]. Specifically, induced p53 activity was significantly higher in *p53^LSL/−^Mdm2^−/−^Mdm4^−/−^* MEFs than that in *p53^LSL/−^Mdm2^−/−^Mdm4^+/−^* MEFs, providing evidence that Mdm4 has an Mdm2-independent role in inhibiting the p53 activity [26] (Figure 2). However, Mdm4 overexpression in mice failed to rescue *Mdm2-null* embryonic lethality [27,28], whereas a modest increase in Mdm2 levels in mice completely rescued *Mdm4^−/−^* embryonic lethality [29]. To further support this observation, seven days of tamoxifen treatment to induce p53 in *Mdm2^−/−^p53^KI/−^* (*Mdm2^−/−^p53ER^TAM^*) mice resulted in lethality within 10 days after treatment, whereas induction of p53 in *Mdm4^−/−^p53^KI/−^* mice did not cause lethality [6,30]. These results suggested that Mdm4’s ability to inhibit p53 activity was considerably less potent than that of Mdm2. 

Although most studies have focused on the p53-dependent functions of MDM4, increasing evidence indicates that MDM4 also has p53-independent functions [31]. However, only a few studies have addressed whether p53-independent functions of MDM4 are dependent or independent of MDM2. Therefore, this review primarily summarizes the p53-independent and MDM2-dependent or -independent functions of MDM4.

## 2. p53-Independent Functions of MDM4

MDM4 is overexpressed in approximately 20% of different types of human cancers, mainly by gene amplification and other unknown mechanisms [7]. Intriguingly, even in tumors that overexpress MDM4, p53 mutations are still observed, although their frequency varies widely across cancer types—from about 6% (hepatocellular carcinoma) to as high as 90% (ovarian serous cystadenocarcinoma) [32,33]. 

For example, in colon cancer patients with p53 mutations, high MDM4 expression is associated with reduced overall survival [33]. These findings suggest that MDM4 contributes to tumor progression through p53-independent mechanisms when overexpressed.

In *Mdm4^Tg15^p53^−/−^* male mice, Mdm4 overexpression (mediated by *Zp3-Cre*) in the *p53^−/−^* background accelerated tumor progression and reduced survival [34,35], suggesting that MDM4 has p53-independent oncogenic functions when overexpressed. Moreover, *Sox2-Cre* recombinase-induced Mdm4 during embryonic development resulted in lethality with severe vascular defects, which was not rescued by p53 deletion [27], further supporting the p53-independent function of Mdm4. This section focuses on the literature associated with MDM4 in the p53-deficient context, in which dependency on MDM2 is not yet well characterized (Table 1). 

**Figure 3:**
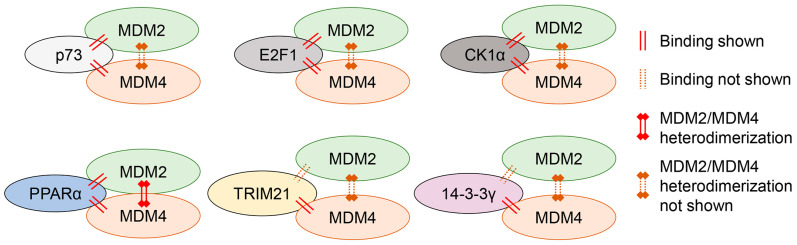
p53-independent functions of MDM4. MDM4 binds to several proteins independently of p53, and some of these proteins also interact with MDM2. However, apart from PPARα, whether MDM4’s effects on modulating the functions of these proteins depend on MDM2 remains unexplored.

MDM4 was reported to promote cell cycle progression independent of p53 (Figure 3). Similarly to MDM2, MDM4 bound to and stabilized TAp73 in p53-null H1299 cells, and knockdown of MDM4 inhibited cell cycle progression, likely due to decreased E2F1 protein levels [36,37]. However, MDM4 has also been shown to bind directly to E2F1 in vitro and to inhibit the Dp-1-enhanced DNA-binding activity of E2F1 when Dp-1 is added to the reaction [38], suggesting that MDM4-mediated inhibition of E2F1 may impair cell cycle progression. This apparent discrepancy regarding the role of MDM4 in regulating E2F1 activity and cell cycle progression suggests that the outcomes of MDM4 modulation may depend on specific experimental conditions and the cellular context. Nonetheless, MDM4 plays a crucial role in cell cycle progression in a manner independent of p53, through its interaction with TAp73 and E2F1. 

Recently, Ueda et al. [39] demonstrated that Mdm4 overexpression (*Mdm4^Tg^*) accelerated AML progression in three different pre-leukemic mouse models (*PU.1 URE^−/−^, Tet2^−/− or +/−^,* and *Flt3^+/ITD^*). MDM4 interacted with casein kinase 1α (CK1α) in p53-null HL60 cells, promoting the nuclear entry and accumulation of β-catenin. Consistent with this mechanism, Mdm4 overexpression in *p53^−/−^* mice caused an expansion of the hematopoietic stem cell (HSC) compartment with activation of the Wnt/β-catenin pathway, indicating that Mdm4 can drive HSC self-renewal and pre-leukemic progression independent of p53. However, whether MDM4 exerts its role on CK1α in complex with MDM2 or independently of MDM2 remains unclear and requires further investigation. 

Intriguingly, MDM4 plays a context-dependent role in ferroptosis. Venkatesh et al. [40] reported that MEL23, an inhibitor of the E3 ubiquitin ligase activity of the MDM2/MDM4 heterodimer, as well as knockdown of MDM2/MDM4, inhibited ferroptosis in p53-knockout (*p53KO*) SK-Hep1 and HT1080 cells and in patient-derived glioblastoma models. These results suggest that the MDM2-MDM4 heterodimer facilitates ferroptosis in a manner independent of p53. Given that MDM2 is reported to regulate the PPARα activity, a key factor in lipid homeostasis, the MDM2/MDM4 heterodimer may facilitate ferroptosis by promoting ubiquitination of PPARα (but not its degradation). Supporting this model, ferroptosis inhibition by MEL23 or by MDM2/MDM4 knockdown was abolished in the presence of a PPARα antagonist (GW6471) in p53-deficient cells. However, the detailed mechanism by which the MDM2/MDM4 complex inhibits PPARα activity to promote ferroptosis remains to be clarified through future studies.

On the other hand, Liu et al. [33] recently reported that MDM4 inhibited ferroptosis by increasing the protein stability of GPX4, an enzyme that reduces lipid peroxidation, in p53-mutated colon cancer cell lines (HT29, SW480). MDM4 upregulated the E3 ubiquitin ligase TRIM21 to promote K63-linked polyubiquitination of GPX4. These K63-linked ubiquitin chains did not target GPX4 for proteasomal degradation but rather modified cellular signaling and protein–protein interactions. Hence, they concluded that K63-polyubiquitinated GPX4 mediated by the MDM4-TRIM21 axis may stabilize GPX4 and thereby inhibit ferroptosis. This may contribute to increased proliferation and tumor growth of p53-mutated colon cancer cells by MDM4 overexpression. However, this study did not address whether the observed MDM4 function depends on MDM2. The observed differences in ferroptosis regulation by MDM4 between the two groups are likely attributable to variations in the cell lines and experimental conditions employed.

Recently, MDM4 has been implicated in glycolytic reprogramming in p53-mutated hepatocellular carcinoma [41]. In Huh7 (p53^Y220C^) cells, MDM4 bound to 14-3-3γ, promoting its cytoplasmic localization and stabilization. The resulting loss of FOXO1 transcriptional activity decreased expression of PCK1 (a key gluconeogenic enzyme) and increased RPIA (a pentose phosphate pathway enzyme), thereby shifting metabolism toward glycolysis. Functionally, this metabolic reprogramming enhanced glucose uptake, lactate secretion, and ATP production, fueling tumor growth under p53-mutated conditions. Although this novel role of MDM4 appears p53-independent, its dependency on MDM2 and missense mutant p53 (mutp53) remains unclear. 

Other p53-independent oncogenic functions of MDM4 (not listed in Table 1) have been examined in p53-knockout HT1080 cells, where MDM4 knockdown or pharmacological inhibition of the MDM2/MDM4 complex by MEL23 reduced migration and focal adhesion [42]. The decrease in these malignant properties was mainly caused by increased protein levels of Spry4 (Sprouty RTK Signaling Antagonist 4) following MDM2 inhibition, as concomitant knockdown of Spry4 reversed these cellular phenotypes. However, MDM2 did not bind to Spry4. Thus, the exact mechanism by which inhibition of the MDM2/MDM4 complex increases Spry4 protein levels has not been fully elucidated and warrants further investigation. 

In contrast to these findings, a report by Matijasevic et al. [43] suggests a tumor suppressive role of Mdm4 in *p53^−/−^* MEFs and mice. Mdm4 deletion in *p53^−/−^* MEFs increased proliferation, immortalization potential, and cellular transformation after passage seven, accompanied by multipolar spindles and reduced chromosome numbers. Moreover, deletion of both *Mdm4* alleles in *p53^−/−^* mice significantly accelerated spontaneous tumor formation compared with *p53^−/−^* mice. These findings highlight a p53-independent role for MDM4 in suppressing malignant progression by regulating centrosomal clustering and chromosome ploidy [43,44]. Since this tumor-suppressive function of MDM4 contradicts most reports describing its oncogenic role, additional studies are needed to delineate whether MDM4 acts as an oncogene or tumor suppressor in the absence of p53.

## 3. p53- and MDM2-Independent Functions of MDM4

**Figure 4:**
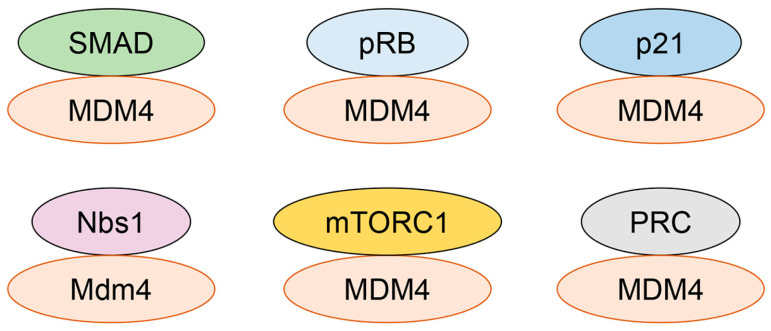
p53- and MDM2-independent functions of MDM4. MDM4 interacts with several proteins independently of p53 and MDM2, indicating functions beyond the canonical p53–MDM2 axis.

Recent studies have increasingly highlighted the function of MDM4 independent of both p53 and MDM2 by using systems such as p53/MDM2 double-null cells and MDM4 deletion mutants lacking both the p53- and MDM2-binding domains (Table 2). The first of these studies demonstrated that MDM4 inhibited the transcriptional activity of SMAD3 and SMAD4 using various MDM4 deletion mutants (Figure 4). MDM4 mutants lacking the N-terminal p53-binding domain (MDM4Δp53), the RING finger domain that does not interact with MDM2 (MDM4ΔRF), or both (MDM4Δp53ΔRF), each suppressed SMAD3/4-dependent transcription without affecting SMAD protein levels. Amino acids 128–444 of MDM4 were found to be critical for its ability to inhibit SMAD3-induced transcriptional activity. MDM4 overexpression also inhibited SMAD3-driven transcription in *p53^−/−^* and *p53^−/−^Mdm2^−/−^* MEFs. Moreover, MDM4 was shown to directly bind SMAD3 and SMAD4 in vitro. These results strongly suggest that the inhibitory effect of MDM4 on SMAD3/4 activity is independent of both p53 and MDM2 [45]. 

Uchida et al. [46] demonstrated that MDM2 bound to the tumor suppressor retinoblastoma protein (pRB), promoting its ubiquitination and subsequent degradation. Later, they found that MDM4 also bound to pRB and inhibited MDM2-mediated ubiquitination of pRB in U2OS and HCT116 cells, as well as in *p53^−/−^Mdm2^−/−^* MEFs. An MDM4 deletion mutant lacking MDM2-binding ability (MDM4ΔC) also retained the capacity to bind the C-terminal region of pRB and inhibit MDM2-mediated ubiquitination of pRB [47]. Moreover, MDM4’s binding to pRB competitively inhibited MDM2-pRB interaction and therefore stabilized pRB protein, leading to restored flat cell formation that had been inhibited by MDM2-mediated reduction of pRB. These results suggest that MDM4 binds to pRB in a manner independent of both MDM2 and p53, where MDM4 competes with MDM2 for pRB binding, contributing to pRB stabilization and activation. 

Related to the role of MDM4 in regulating cell cycle components, Jin et al. [48] reported that MDM4 reduced protein levels of p21 in HEK293 cells, acting in cooperation with MDM2. Interestingly, this reduction was also observed in *p53^−/−^Mdm2^−/−^* MEFs following MDM4 overexpression, and direct binding between MDM4 and p21 was confirmed using in vitro synthesized proteins, indicating an MDM2-independent mechanism. As expected, MDM4 depletion resulted in p21-mediated G1 arrest in H1299 cells. Mechanistically, MDM4 promoted proteasomal degradation of p21 in a manner independent of protein ubiquitination by forming a complex with the S2 subunit of the 26S proteasome. However, the detailed mechanism remains unclear. 

By analyzing The Cancer Genome Atlas (TCGA) database, the Eischen’s group [32] found that p53 mutations or deletions persist despite MDM4 overexpression in tumors. They further demonstrated that overexpression of Mdm4, and even a mutant Mdm4 lacking the RING finger domain (ΔRING), inhibited DNA double-strand break (DSB) repair and increased chromosome aberrations in *p53^−/−^Mdm2^−/−^* MEFs, similar to effects observed with Mdm2 overexpression. Both Mdm4 and its ΔRING mutant interacted with Nbs1/Nibrin, providing a mechanism by which Mdm4 inhibits DSB repair independent of p53 and Mdm2. This interaction rapidly increased in the chromatin-bound fraction following DNA damage and decreased as DNA was repaired, highlighting a novel role for Mdm4 in regulating the DNA repair pathway [32].

MDM4 has also been shown to bind cytoplasmic mTOR and inhibit mTOR kinase activity and downstream mTORC1 signaling, leading to reduced cell size and proliferation in an mTOR-dependent manner [49]. Deletion of *Mdm4* (*p53^−/−^Mdm4^−/−^*) in *p53^−/−^* MEFs significantly increased pS6K1 levels compared with *p53^−/−^* MEFs, whereas deletion of *Mdm2* in *p53^−/−^* MEFs (*p53^−/−^Mdm2^−/−^*) did not alter pS6K1 levels. This result suggested a potential p53- and MDM2-independent role for MDM4 in restraining mTORC1 signaling. However, experiments altering Mdm4 expression were not performed in *p53^−/−^Mdm2^−/−^* MEFs to conclusively establish p53- and Mdm2-independence. Moreover, MDM4 knockdown in MDA-MB-231 cells resulted in increased mammosphere formation, which was nullified by rapamycin treatment [49]. Intriguingly, high levels of *MDM4* mRNA correlated with low *mTOR* mRNA expression in breast cancers carrying p53 mutations. These observations suggested a possible tumor-suppressive role for MDM4 in the absence of functional p53, consistent with a previous report showing that high MDM4 expression correlated with favorable clinicopathological features in breast cancer patients in a manner independent of p53 [50]. Thus, in the absence of functional p53, MDM4 may inhibit tumor progression by suppressing mTORC1 signaling. It remains unclear under which experimental conditions or cellular contexts MDM4 functions as an oncogene or a tumor suppressor.

**Table 2:** p53- and MDM2-independent functions of MDM4.

Proteins	Interactions	Outcomes	References
SMAD3/4	MDM4 binds to SMAD3 and SMAD4 in vitro and in H1299 cells.	MDM4 inhibits the transcriptional activity of SMAD proteins.	[45]
pRB (Retinoblastoma protein)	MDM4 binds to pRB in *p53^−/−^Mdm2^−/−^* MEFs	MDM4’s binding to pRB increases pRB protein levels, leading to increased flat cell formation.	[47]
p21	MDM4 binds to p21 in vitro and in H1299 cells.	MDM4 enhances the proteasomal degradation of p21, whereas its depletion increases G1 cell cycle arrest in p*53^−/−^Mdm2^−/−^* MEFs and H1299 cells in a manner dependent on p21.	[48]
Nbs1/ Nibrin	Mdm4 binds to Nbs1 in *p53^−/−^Mdm2^−/−^* MEFs.	Mdm4 inhibits DSB repair and increases chromosome and chromatid breaks, thereby promoting chromosomal instability.	[32]
mTORC1	MDM4 binds to mTOR in HeLa and HEK293T cell lines.	Deletion of *Mdm4* in *p53^−/−^* MEFs increases mTOR activity, leading to elevated phosphorylation of the mTOR downstream target p70S6K1, a change not observed upon deletion of *Mdm2* in *p53^−/−^* MEFs. MDM4 knockdown enhances mammosphere formation, which is nullified by rapamycin.	[49]
PRCs (RNF2, EZH2)	MDM4 binds to RNF2, a PRC1 member, and EZH2, a PRC2 member, in H1299 cells.	MDM4 supports DNA replication fork progression in *p53^−/−^Mdm2^−/−^* MEFs, as deletion of *Mdm4 in p53^−/−^Mdm2^−/−^* MEFs (*p53^−/−^Mdm2^−/−^Mdm4^−/−^*) further reduces replication fork progression. Moreover, MDM4 depletion sensitizes H1299 cells to gemcitabine.	[51]

Recently, MDM4 has been reported to play a role in DNA replication similar to MDM2 [51]. Depletion of MDM4 impaired replication fork progression and induced R-loops (DNA/RNA hybrids), major obstacles to replication, in p53-deficient cells. Co-depletion of MDM2 and MDM4 in H1299 cells and *p53^−/−^* MEFs cooperatively decreased replication fork progression compared with single depletions, indicating their cooperative yet independent roles in DNA replication. Overexpression of MDM4 partially restored replication fork progression in *p53^−/−^Mdm2^−/−^* and *p53^−/−^Mdm2^−/−^Mdm4^−/−^* MEFs, further confirming the p53- and MDM2-independent function of MDM4 in regulating DNA replication. Mechanistically, MDM4 bound RNF2, a PRC1 member, and EZH2, a PRC2 member, thereby supporting replication fork progression. To reinforce these observations, MDM4 depletion sensitized H1299 cells to gemcitabine, a nucleoside analog that induces replication stress. Thus, MDM4 plays a role in promoting DNA replication fork progression independent of p53 and MDM2. However, the precise mechanism by which MDM4’s interactions with RNF2 and EZH2—core components of PRC1 and PRC2, respectively—promote replication fork progression remains unclear, as the study did not determine whether MDM4 alters the chromatin-modifying activity of PRC1/PRC2 or affects local chromatin accessibility at replication forks.

## 4. Conclusions

This review article primarily focuses on the p53-independent functions of MDM4, some of which are also independent of MDM2. Because MDM4 lacks intrinsic E3 ubiquitin ligase activity, unlike MDM2, it may modulate the recruitment, stabilization, or destabilization of various protein complexes through its interactions with target proteins. Structure-based analysis of MDM4 in p53- and MDM2- independent context has not yet been fully characterized. While one study reported that MDM4 can form homodimers in living cells using eGFP-tagged constructs [52], others suggest that MDM4 does not form homodimers under physiological conditions [53]. Clarifying whether MDM4 functions as a monomer or homodimer independent of MDM2 and identifying the domains responsible for MDM4-target protein interactions are critical for defining its p53- and MDM2-independent functions. 

Despite extensive research on the MDM4–p53 axis, little attention has been given to whether MDM4 regulates the gain-of-function (GOF) activity of missense mutp53, although MDM4 overexpression is shown to promote proliferation and tumor growth in p53-mutated colon cancer cells [33]. Further studies are required to elucidate MDM4’s role in mutp53 GOF and the underlying mechanism, including MDM2’s involvement in the MDM4-mutp53 GOF axis.

*MDM2/MDM4* gene amplification has emerged as a potential genomic marker associated with hyperprogression of cancer following treatment with immune checkpoint inhibitors (ICIs). In a study of 155 stage IV cancer patients treated with PD-1/PD-L1 or CTLA-4 inhibitors, six patients harboring *MDM2/MDM4* amplification experienced treatment failure with dramatic increases in tumor burden, highlighting the importance of genomic profiling to identify patients at risk for hyperprogression [54]. Because these analyses did not identify p53 mutations, the observed ICI resistance is likely independent of p53 status. Further studies are needed to elucidate the underlying mechanisms by which MDM2 and MDM4 contribute to ICI resistance and cancer hyperprogression, as well as their functional association with p53. Importantly, the role of MDM4 in cancer immunity remains poorly understood. Liu et al. [55] reported a positive correlation between MDM4 overexpression and tumor immune infiltration through analyses using multiple algorithms based on TCGA database and confirmed immune signaling activation by MDM4 overexpression in a p53-mutated SW480 cell line via RNA sequencing. They demonstrated that MDM4 overexpression activates multiple immune response pathways, including type I interferon signaling, cytokine–receptor interactions, chemokine signaling, and NF-κB–related inflammatory programs. Functionally, MDM4 promoted macrophage migration and favored M2-like polarization in co-culture assays, suggesting that MDM4 can directly influence components of the tumor immune microenvironment. Future studies should determine whether these immunomodulatory activities of MDM4 contribute to ICI resistance or hyperprogression in a manner dependent on p53 and/or MDM2, as well as the underlying molecular mechanisms in greater detail.

Notably, although emerging evidence has identified several p53- and MDM2-independent oncogenic functions for MDM4, therapeutic strategies for MDM4-overexpressing cancers have largely focused on p53 reactivation (e.g., ALRN-6924). To the best of our knowledge, no preclinical or clinical agents have been developed that specifically target MDM4-driven tumors harboring p53 mutations or deletions. Identifying compounds that induce cytotoxicity selectively in MDM4-overexpressing cancers, independently of p53 status, will therefore be an important direction for future research.

In conclusion, current knowledge establishes MDM4 as a regulator of diverse signaling through both p53- and/or MDM2-dependent and -independent mechanisms. However, many mechanistic aspects remain unresolved, including (1) the possible role of MDM4 in mutp53 regulation, (2) its reliance on MDM2, (3) structural determinants of its protein interactions, (4) its context-dependent oncogenic or tumor suppressive function in the absence of p53, and (5) its involvement in modulating tumor immunity and hyperprogression of cancer following ICI treatment. Addressing these questions will refine our mechanistic understanding of MDM4 functions and may inform the development of therapeutic strategies directed at MDM4-overexpressing tumors. Considering the lack of enzymatic activity in MDM4, approaches that prevent its interaction with specific binding partners may provide selective and effective therapeutic avenues.

## Figures and Tables

**Table 1:** p53-independent functions of MDM4.

Proteins	Interactions	Outcomes	References
TAp73	Both MDM4 and MDM2 bind to TAp73 to stabilize TAp73 in HEK293 or H1299 cells.	Knockdown of MDM4 lowers TAp73 protein levels, resulting in reduced proliferation of H1299 cells.	[36,37]
E2F1	MDM4 binds to E2F1 in vitro and in Saos2 cells (E2F1 is also known to bind MDM2).	MDM4 inhibits the Dp-1-mediated DNA-binding ability of E2F1.	[38]
Casein Kinase 1 α (CK1α)	MDM4 binds to CK1α in HL60 cells (CK1α also binds to MDM2).	Mdm4 overexpression causes an expansion of the HSC compartment with activation of the Wnt/β-catenin pathway and drives pre-leukemic progression in *p53^−/−^* mice.	[39]
PPARα	Both MDM2 and MDM4 associate with PPARα in *p53KO* SK-Hep1 and HT1080 cells.	MEL23, inhibitor of the E3 ubiquitin ligase activity of the MDM2/MDM4 heterodimer, suppresses ferroptosis in *p53KO* cells in a manner dependent on PPARα. The MDM2/MDM4 complex promotes ferroptosis, likely by facilitating ubiquitination (but not degradation) of PPARα.	[40]
TRIM21	MDM4 binds to TRIM21 in HT29 colorectal cancer cells (p53^R273H^).	MDM4 increases protein stability of GPX4, an enzyme that reduces lipid peroxidation, by inhibiting TRIM21-mediated ubiquitination and degradation of GPX4, thereby contributing to the suppression of ferroptosis. This may contribute to increased proliferation and tumor growth of p53-mutated colon cancer cells by MDM4 overexpression.	[33]
14-3-3γ	MDM4 binds to 14-3-3γ in Huh7 cell line.	MDM4 promotes cytoplasmic localization of 14-3-3γ which binds to FOXO1, trapping FOXO1 in the cytoplasm and accelerating its degradation. This decreases PCK1 expression and elevates RPIA levels, shifting metabolism toward glycolysis.	[41]

## Data Availability

No new data were created or analyzed in this study.
